# Dual-Prep registry: Atherectomy devices and intravascUlAr lithotripsy for the PREParation of heavily calcified coronary lesions registry, 1-year results

**DOI:** 10.1007/s12928-026-01264-4

**Published:** 2026-03-19

**Authors:** Masato Nakamura, Nehiro Kuriyama, Yutaka Tanaka, Seiji Yamazaki, Tomohiro Kawasaki, Takashi Muramatsu, Kazushige Kadota, Takashi Ashikaga, Akihiko Takahashi, Satoru Otsuji, Kenji Ando, Masaru Ishida, Shigeru Nakamura, Yoshiaki Ito, Raisuke Iijima, Gaku Nakazawa, Junya Shite, Junko Honye, Junya Ako, Hiroyoshi Yokoi, Ken Kozuma, Hiromasa Otake, Kenji Kochi, Tomomi Yamada, Yohei Sotomi

**Affiliations:** 1 Division of Minimally Invasive Treatment in Cardiovascular Medicine, Toho University Ohashi Medical Center, 2-22-36, Ohashi, Meguro-Ku, Tokyo, 153-8515 Japan; 2 Department of Cardiology, Miyazaki Medical Association Hospital, Miyazaki, Japan; 3 Department of Cardiology, Shonan Kamakura General Hospital, Kanagawa, Japan; 4 Department of Cardiology, Sapporo Higashi Tokushukai Hospital, Hokkaido, Japan; 5 Department of Cardiology, Tenjinkai Shin-Koga Hospital, Fukuoka, Japan; 6 Department of Cardiology, Fujita Health University Hospital, Aichi, Japan; 7 Department of Cardiology, Kurashiki Central Hospital, Okayama, Japan; 8 Department of Cardiology, Japanese Red Cross Musashino Hospital, Tokyo, Japan; 9 Department of Cardiology, Sakurakai Takahashi Hospital, Hyogo, Japan; 10 Department of Cardiology, Higashi Takarazuka Satoh Hospital, Hyogo, Japan; 11 Department of Cardiology, Kokura Memorial Hospital, Fukuoka, Japan; 12 Division of Cardiology, Department of Internal Medicine, Iwate Medical University, Iwate, Japan; 13 Department of Cardiology, Kyoto Katsura Hospital, Kyoto, Japan; 14 Department of Cardiology, Saiseikai Yokohamashi Tobu Hospital, Kanagawa, Japan; 15 Department of Cardiovascular Medicine, Toho University Ohashi Medical Center, Tokyo, Japan; 16 Department of Cardiology, Kindai University Hospital, Osaka, Japan; 17 Department of Cardiology, Osaka Saiseikai Nakatsu Hospital, Osaka, Japan; 18 Department of Cardiology, Kikuna Memorial Hospital, Kanagawa, Japan; 19 Department of Cardiovascular Medicine, Kitasato University Hospital, Kanagawa, Japan; 20 Cardiovascular Center, Fukuoka Sanno Hospital, Fukuoka, Japan; 21 Department of Cardiology, Teikyo University Hospital, Tokyo, Japan; 22 Division of Cardiovascular Medicine, Kobe University Graduate School of Medicine, Hyogo, Japan; 23 Department of Medical Innovation, The University of Osaka Hospital, Osaka, Japan; 24 Department of Cardiovascular Medicine, The University of Osaka Graduate School of Medicine, Osaka, Japan

**Keywords:** Intravascular lithotripsy, Atherectomy, Calcified lesion, Calcium nodule

## Abstract

Combination therapy with atherectomy and intravascular lithotripsy (IVL) has emerged as a promising strategy for the treatment of severely calcified occlusive coronary lesions, which potentially enhances procedural efficacy without increasing complication risk. The Dual-Prep Registry is a multicenter, prospective registry designed to evaluate the safety and efficacy of IVL after atherectomy in severely calcified lesions. Combined use was selectively applied when the risk of complications was anticipated to be high with a larger atherectomy burr size, or when it was deemed non-beneficial due to unfavorable guidewire bias. All adverse events were adjudicated by a clinical events committee. Kaplan–Meier analysis was performed to evaluate the primary endpoint of major adverse cardiovascular events (MACE; composite endpoint of cardiac death, myocardial infarction, and target vessel revascularization [TVR]) at 1 year. A total of 118 cases (120 lesions) were enrolled across 20 facilities. Significant comorbidities included diabetes in 56.8% of patients and hemodialysis-dependence in 25.4%. Calcification score after atherectomy was 4.0 in all cases, and calcified nodules were present in 41.7% (core-lab analysis) of cases. One-year follow-up was complete in 99.2% patients. MACE occurred in 7.6% patients at one year (cardiac death 2.5%, myocardial infarction 5.1%, TVR 5.1%) and stent thrombosis was observed in 1 case. Atherectomy followed by IVL resulted in low 1-year rates of MACE, TVR, and stent thrombosis in patients with severely calcified coronary lesions. This approach may be considered for lesions where an “IVL-first” strategy is difficult to apply. Japan Registry of Clinical Trials: jRCT1032230384. URL: https://jrct.mhlw.go.jp.

**Graphical Abstract:**

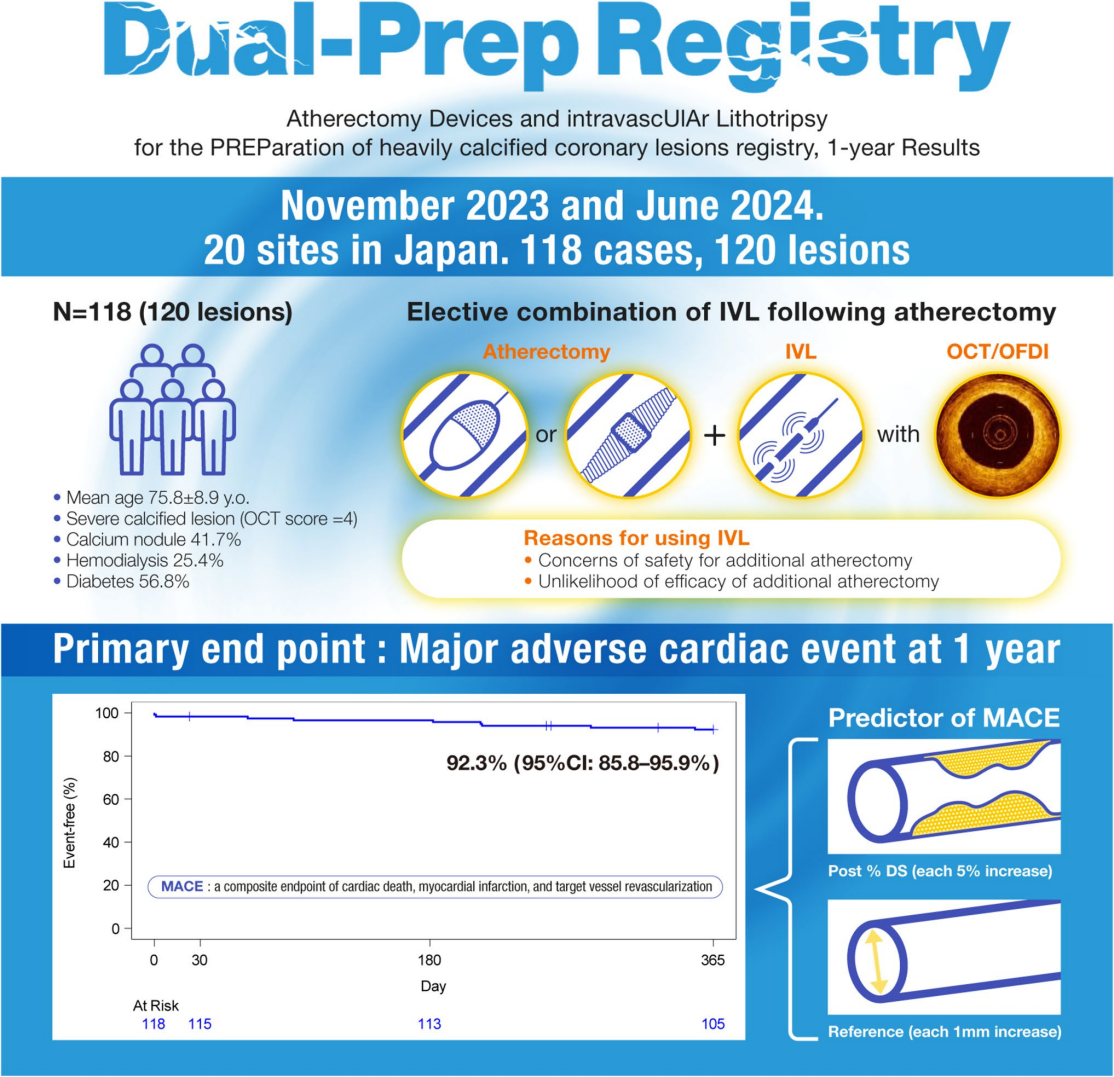

**Supplementary Information:**

The online version contains supplementary material available at 10.1007/s12928-026-01264-4.

## Introduction

Severely calcified coronary lesions pose a significant barrier to successful percutaneous coronary intervention (PCI). These lesions affect 10–25% of cases [1] and lead to device delivery failure, poor stent expansion, increased procedural complications, and adverse long-term outcomes, including restenosis and thrombosis. Several devices have been developed to address calcified lesions, including rotational atherectomy (RA), orbital atherectomy (OA), and intravascular lithotripsy (IVL), each of which uses a distinct mechanism of action. Despite demonstrated procedural benefits, however, no significant difference in long-term outcomes among these devices has been observed, and no gold-standard strategy for severely calcified lesions has been established. Recent evidence is inconclusive. The ECLIPSE trial compared OA with balloon dilatation for lesion preparation in 2,005 cases of highly calcified lesions, but found no significant difference in 1-year target vessel failure rates (11.5% vs. 10.8%).[2] Furthermore, studies using angiographic guidance have produced inconsistent findings, suggesting that angiography is insufficient for evaluating calcification severity and that intravascular imaging guidance is crucial for optimizing treatment strategies [3].

Given the inherent advantages and limitations of each standalone device, combination therapy using atherectomy followed by IVL has emerged as a promising strategy that may enhance efficacy while mitigating individual device limitations. However, prospective data on long-term outcomes of this combined approach remain scarce. The critical question is whether favorable short-term outcomes observed with combination atherectomy-IVL therapy are sustained over the long term. If such benefits persist, this approach could represent a valuable treatment option for lesions which are unsuitable for IVL-first strategies. Conversely, if early benefits are not maintained, the added procedural complexity may not be justified. Thus, establishing the durability of combination therapy outcomes remains essential for clinical decision-making.

Here, we report 1-year outcomes from the Dual-Prep registry, a prospective, multicenter study which evaluated the safety and efficacy of adjunctive IVL following atherectomy in patients with severely calcified coronary lesions confirmed by intravascular imaging. [4] This analysis extends our previously reported favorable 30-day procedural outcomes and optimal stent expansion results to assess whether these benefits translate into sustained clinical benefit at one year. Of note, a substantial proportion of the severely calcified lesions in the present cohort were calcified nodules, a particularly challenging lesion phenotype associated with poor prognosis.

### Study design

**Fig. 1:**
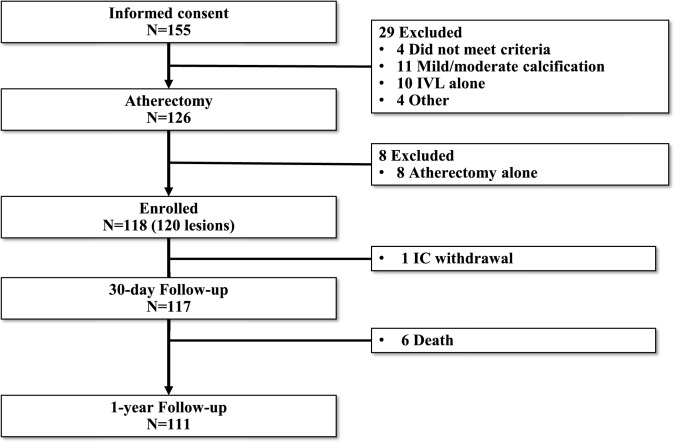
Study flow chart. Patients were enrolled from 20 sites in Japan through November 2023 to June 2024. 1 year follow-up rate was 99.2%. IVL: intravascular lithotripsy

DUAL-PREP is a prospective, multicenter, single-arm study designed to assess the safety and efficacy of combination use of atherectomy devices and IVL before DES deployment in patients with severely calcified lesions. Severity of calcification was initially evaluated angiographically. A calcification score of 3 or greater prior to IVL was a mandatory requirement and was subsequently confirmed by intravascular imaging. [5] The details of the study protocol have been published previously.[4] In brief, enrollment required: 1) age 18 years or older; 2) consent to participate in the study; and 3) the presence of severely calcified lesions for which treatment with IVL after other atherectomy devices may be preferable, based on imaging findings. Exclusion criteria included: 1) participation in other clinical trials that may affect the results; and 2) ineligibility for treatment with atherectomy or IVL. The study protocol was approved by central review (Institutional Review Board of Toho University Ohashi Hospital Ethics Board H23024 H23 May 22, 2023). A flow chart of the present study is shown in Fig. 1. RA/OA was performed at the operator’s discretion in a standard fashion. [6] After RA/OA, IVL was used when a calcified lesion was considered inadequately pretreated but further atherectomy was inappropriate, e.g. slow flow, deep calcification, or when guidewire bias limited atherectomy effectiveness. Finally, DES was deployed after IVL and any subsequent postdilatation.

### Study endpoints

The primary safety end points of this study were 30-day major adverse cardiac events (MACE) and procedural success, each of which has been reported in previous study. [4] Additional prespecified clinical analysis at 1 year in this study included MACE (a composite of cardiovascular death, myocardial infarction (MI), or clinically driven target vessel revascularization (CD-TVR)), target lesion revascularization (TLR), MI (Fourth universal definition) [7], and target lesion failure (TLF; a composite of cardiac death, MI, TLR). Angiograms and intravascular imaging (OCT/OFDI) were independently adjudicated by a core lab (Micron, Inc, Osaka, Japan), and clinical events were independently adjudicated by a Clinical Events Committee (CEC).

### Statistical analysis

Continuous variables were expressed as mean ± SD. Categorical variables were summarized as frequencies and proportions. Kaplan–Meier analysis was performed to estimate cumulative event-free rate of MACE, TLF, and component of MACE at 1-year follow-up. 95% confidence interval (CI) was calculated by Greenwood’s formula. Furthermore, Brookmeyer and Crowley’s method was used to calculate the CI for median survival time. Factors associated with MACE were evaluated using univariate logistic regression analysis with Firth’s penalized likelihood method. All tests were two-sided, and p values < 0.05 were considered statistically significant. Analyses were performed by an independent biostatistician using SAS version 9.4 statistical analysis software (SAS Institute Inc., Cary, NC, USA).

## Results

### Subjects and procedure

Informed consent was obtained from 156 patients at 20 participating centers between November 2023 and June 2024. From this cohort, 28 cases that did not undergo atherectomy and 8 cases that underwent atherectomy alone without concomitant IVL were not registered in this registry. The primary reason for exclusion was insufficient degree of calcification severity on intravascular imaging rather than failure to meet the registration criteria. Consequently, 118 cases (120 lesions) were analyzed in this registry. All patients were enrolled at the time of attempted treatment with the IVL system after atherectomy, regardless of whether the IVL catheter reached the lesion or not. A flow chart of the present study is shown in Fig. 1.

Patient demographics and lesion characteristics are depicted in Table 1. Mean age was 75.8 ± 8.9 years, 70.3% of patients were male, 56.8% had diabetes mellitus and 25.4% were on hemodialysis. 91.5% had de novo coronary artery lesions and were primarily patients with chronic coronary syndrome (89.8%). The principal treated vessel was the left anterior descending artery (64.2%) and mean reference vessel diameter was 2.67 ± 0.69 mm, with mean lesion length 34.3 ± 15.2 mm. Procedural factors are shown in Table 2. A transradial approach was applied in 63.5% of cases. RA was performed in 83.9% of patients with a mean burr size of 1.57 ± 0.20 mm. The remaining 16.9% underwent OA. The calcification score of lesions after atherectomy remained at 4.0 in all cases. Additional IVL after atherectomy was performed in 42.4% of cases due to safety concerns; in 60.2% of cases because additional atherectomy was not expected to be effective; and in 1.7% of cases for other reasons (multiple responses allowed). In all patients, the IVL was successfully traversed to the target lesion site. The IVLs were primarily 2.5 mm and 3.5 mm in diameter, and used in 36.7% and 44.2% of cases, respectively. A DES was implanted in all cases, with a mean diameter of 3.19 ± 0.51 mm and length of 36.3 ± 16.0 mm. DES postdilatation was performed in 79.2% of cases, with a mean maximum balloon diameter of 3.45 ± 0.58 mm mm and mean inflation pressure of 17.9 ± 4.6 atmospheres. Procedural success was achieved in 117/120 lesions (97.5%; 92.9–99.5). Following PCI, 68 patients (57.6%) continued dual antiplatelet therapy for one year, while 24.6% were prescribed P2Y12 inhibitor monotherapy (Figure. S1).

**Table 1:** Patient demographics and lesion characteristics

Patient demographics n = 118 (%)	
Age (years)	75.8 ± 8.9
Male	83 (70.3)
Body weight (kg)	60.3 ± 12.9
Body mass index	23.3 ± 3.9
Diabetes mellitus	67 (56.8)
Hypertension	97 (82.2)
Hyperlipidemia	91 (77.1)
History of smoking	52 (44.1)
Clinical presentation	
Stable angina pectoris	80 (67.8)
ACS	12 (10.2)
Silent ischemia	26 (22.0)
Previous myocardial infarction	21 (17.8)
Previous stroke	17 (14.4)
Previous PCI	44 (37.3)
History of coronary artery bypass graft	4 (3.4)
Atrial fibrillation	17 (14.4)
De novo lesion	108 (91.5)
Ejection fraction	57.0 ± 10.7
eGFR	45.1 ± 27.0
Hemodialysis	30 (25.4)
Lesion characteristics n = 120 (%)	
Lesion location	
Right coronary artery	37 (30.8)
Left descending artery	77 (64.2)
Left circumflex artery	4 (3.3)
Left main trunk*	6 (5.0)
Chronic total occlusion	2 (1.7)
Bifurcation lesion	31 (25.8)
Calcium nodule	50 (41.7%)
Lesion classification Type B2/C	120 (100)
Reference vessel diameter (mm)	2.67 ± 0.69
Minimum lumen diameter (mm)	0.72 ± 0.28
Diameter stenosis (%)	72.6 ± 9.6
Lesion length (mm)	34.3 ± 15.2

PCI: percutaneous coronary intervention. eGFR: estimated glomerular filtration rate, using the MDRD formula, *Left main trunk includes three lesions for LMT-LAD and one lesion for LMT-LCX

**Table 2:** Procedural characteristics

Characteristic	
Radial approach	75 (63.6)
Transfemoral approach	38 (32.2)
Brachial approach	5 (4.2)
Rotational atherectomy*	99 (83.9)
Step up of burr size	10 (8.5)
Used burr size	
1.25 mm	17 (17.2)
1.5 mm	60 (60.6)
1.75 mm	23 (23.2)
2.0 mm	9 (9.1)
Orbital atherectomy	20 (16.9)
IVL treatment	
Number of catheters per case	1.10 ± 0.30
2.5 mm	44 (36.7)
3.0 mm	53 (44.2)
3.5 mm	23 (19.2)
4.0 mm	12 (10.0)
Total number of pulses	76.5 ± 22.9
Side branch protection	29 (24.2)
Postdilatation before stent deployment	51 (42.5)
Modified balloon	32 (62.7)
Non-compliant balloon	20 (39.2)
Max balloon size (mm)	2.96 ± 0.48
Max dilatation pressure (atm)	16.5 ± 4.8
Drug eluting stent	
Stent diameter (mm)	3.19 ± 0.51
Total stent length (mm)	36.3 ± 16.0
Post-stent dilatation	95 (79.2)
Max balloon size (mm)	3.45 ± 0.58
Max dilatation pressure (atm)	17.9 ± 4.6
OCT/OFDI use as a guidance of PCI	109 (90.8)
IVUS use as a guidance of PCI**	19 (16.5)
Procedural success***	117 (97.5%)

IVL: intravascular lithotripsy, IVUS; intravascular ultrasound, OCT/OFDI: optical coherence tomography/optical frequency domain imaging. *1 case was treated with RA followed by OA. **Both IVUS and OCT/OFDI were applied to guide PCI in 8 lesions. *** Procedural success was defined as residual stenosis < 50% after stenting without MACE during hospitalization (core laboratory assessment)

### 1-year outcomes

**Fig. 2:**
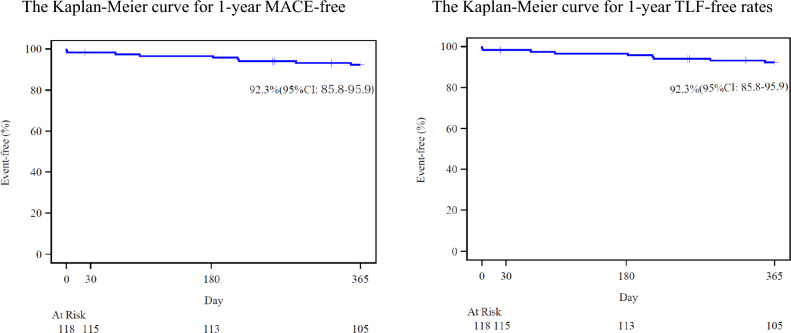
The Kaplan–Meier curve for 1-year MACE-free and TLF-free rates

The 1-year follow-up rate was 99.2%. The Kaplan–Meier estimates for the 1-year MACE-free and TLF-free rates were both 92.3% (95%CI: 85.8–95.9%), and the 1-year TLR-free rate was estimated to be 94.8% (95%CI: 88.9–97.7%) (Fig. 2). The incidence of MACE components and secondary endpoints is shown in Table 3 and Figure S2, respectively. All-cause death occurred in 5.1%, of which 2.5% were cardiac deaths. All TVRs (5.1%) were related to the target lesion. MI occurred in 5.1% of patients, including 1.7% procedure-related MIs. MACE was observed in 5/60 (8.3%) of cases with calcified nodules and 4/49 (8.2%) of those without. Stent thrombosis was observed in one case involving a nonagenarian female with atrial fibrillation. The patient underwent mid-right coronary artery PCI utilizing RA (1.5 mm) and IVL (4.0 mm), followed by deployment of a 4.0 × 18 mm everolimus-eluting stent with post-dilation at 22 atm. Antithrombotic therapy consisted of edoxaban 15 mg, with prasugrel 3.75 mg withdrawal at 2 months. Seven months later, the patient presented with acute coronary syndrome. Angiography confirmed subtotal thrombotic occlusion, which was successfully managed with a drug-coated balloon.

**Table 3:** 1-year outcomes

	n (%)
MACE 1 year	9 (7.6)
Cardiac death	3 (2.5)
MI	6 (5.1)
CD-TVR	6 (5.1)
TLF	9 (7.6)
Stent thrombosis	1 (0.8)
Definite	1 (0.8)
Probable	0 (0)
All death	6 (5.1)
Cardiovascular death	4 (3.4)
Non-CV death	2(1.7)
All MI	6 (5.1)
Periprocedural MI	2 (1.7)
Spontaneous MI	4 (3.4)
All revascularization	9 (7.6)
CD-TLR	6 (5.1)
Non-target-vessel-related TVR	3 (2.5)

MACE: major adverse cardiac event, MI: myocardial infarction, TVR: target vessel revascularization. TLR: target lesion revascularization

### Factors associating with MACE

Table 4 shows a univariate logistic regression analysis for MACE. Actual incidence rates in various subgroups are shown in Table S1. Both % residual stenosis after stent placement and lesions treated in originally larger vessels showed a significant positive association with MACE, while other factors such as procedural characteristics and patient background were not associated with MACE. A 5% increase in residual stenosis and 1-mm increase in reference vessel diameter were each associated with a 2.3-fold increase in MACE. Notably, neither eruptive nor non-eruptive calcified nodules were statistically significant risk factors for MACE at 1 year (p = 0.422 and 0.478, respectively).

**Table 4:** Univariate logistic regression analysis with Firth’s penalized likelihood approach

	OR	95% CI	p-Value
Age (each 5 year increase)	1.53	0.96–2.42	0.072
Male	0.31	0.08–1.18	0.087
Body weight (each 5 kg increase)	0.91	0.70–1.19	0.503
DM	2.46	0.55–10.93	0.239
HL	0.33	0.09–1.27	0.108
Smoking	0.80	0.22–2.98	0.744
Dialysis	2.58	0.68–9.80	0.165
eGFR (each 10 ml/min/1.73m^2^ increase)	0.82	0.64–1.05	0.118
LAD	0.44	0.12–1.65	0.223
Ostial	1.68	0.38–7.53	0.497
Reference (each 1 mm increase)	2.27	1.05–4.90	0.037
Pre MLD (each 1 mm increase)	4.92	0.63–38.6	0.130
Pre % DS (each 5% increase)	1.06	0.75–1.49	0.753
Post MLD (each 1 mm increase)	1.60	0.52–4.89	0.413
Post % DS (each 5% increase)	2.34	1.28–4.30	0.006
Burr to Artery ratio (each 0.1 increase)	0.64	0.35–1.17	0.150
IVL to vessel ratio (each 0.1 increase)	0.91	0.68–1.22	0.522
Stent to vessel ratio (each 0.1 increase)	0.84	0.61–1.15	0.269
Eruptive calcified nodule	1.78	0.43–7.33	0.422
Non-eruptive calcified nodule	0.50	0.07–3.43	0.478
Min stent expansion index (each 10 increase)	1.01	0.64–1.57	0.977
Asymmetry index (each 0.1 increase)	1.08	0.72–1.62	0.708
Min eccentricity index (each 0.1 increase)	0.78	0.40–1.53	0.476
Min stent area (each 1 mm^2^ increase)	1.19	0.92–1.55	0.183

## Discussion

This is the first prospective study to evaluate 1-year outcomes of follow-on use of IVL after atherectomy for severely calcified coronary lesions under intravascular imaging guidance. Results showed that the estimated freedom from MACE at 1 year was 92.3% (95%CI: 85.8–95.9%) and the estimated 1-year TLR-free rate was 94.8% (95%CI: 88.9–97.7%). Further, factors associated with MACE were residual % stenosis and reference vessel diameter. These findings suggest that the combination of IVL with atherectomy may be an effective treatment strategy in cases where IVL-first approaches are difficult to apply.

### Comparison with previous studies using standalone therapy

While numerous reports indicate that the use of lesion preparation devices improves acute procedural success for calcified lesions, long-term outcomes remain inconsistent. For instance, although the ROTAXUS reported improved initial results with RA (procedural success 92.5%), the 9-month event rates were 24.2%. [8] Similarly, the ORBIT II trial for OA reported a 1-year MACE rate of 16.4%. [9] Overall, there are no significant differences in reported 1-year outcomes among these devices. The average MACE rate is 15% (range: 13.2%–26%) for RA [9–11] and 14.4% (range: 11%–16.4%) for OA. [11–13] Although IVL is reported to be slightly lower at 13.2% (range: 9.4%–13.8%), [14, 15] the superiority of IVL over other devices regarding long-term outcomes is not yet evident. Limitations associated with the outcomes of individual devices have recently led to growing interest in combination therapy. In fact, trends in atherectomy and IVL use among PCI patients in the US noted that IVL usage increased from 0.04% to 2.57% between January 2021 and June 2022. [16] Among these cases, only 15.6% met the inclusion or exclusion criteria of the pivotal trials. Cases not meeting the approval trial criteria were primarily acute coronary syndrome cases, in-stent restenosis, and combined treatments with atherectomy. [17] Furthermore, reports from the US CathPCI Registry also indicate that combined therapy has increased over time alongside the rise in IVL use. [18] However, data on the long-term outcomes of combination therapy remain scarce. The DUAL-Prep Registry addresses these limitations. In the present study, the estimated freedom from MACE at 1 year was 92.3%. While this rate appears comparable to previously reported outcomes for the stand-alone strategy, it is important to note that direct comparisons were not performed. The background characteristics and study populations differ substantially between our cohort and those in prior reports, limiting any definitive conclusions regarding relative efficacy. In a study on long-term outcomes reported for 114 cases of combination therapy conducted in Europe from May 2019 to December 2023, the reported one-year MACE of 9% appears consistent with the current results; however, it should be noted that the prior study was retrospective and had a longer registration period (5 years) compared to this trial (8 months). [19] Furthermore, the elective combined approach rate for RA/OA-IVL was 100% in this study versus 57% in the retrospective report, and the one-year follow-up rate was 97% versus 67%. Therefore, the Dual-Prep registry is the first study to demonstrate the 1-year efficacy of combination therapy without these potentially confounding factors, and in a prospective fashion. We therefore consider that the MACE incidence of 7.6% and TLR incidence of 5.1% in this prospective study to be favorable outcomes.

The most likely reasons for the superiority of MACE rate compared to RA alone are likely to include the strategic use of smaller burr-vessel ratios paired with the use of IVL to complete the procedure. This may contribute to a reduction in in-hospital events, including procedural complications. Indeed, this registry evaluated the additive effects of combining RA and IVL in cases where increased burr size after initial RA/OA therapy was considered unlikely to be effective or where a high risk of complications was anticipated. Furthermore, the synergistic effects of this combination strategy also likely contribute to outcomes. Intravascular imaging was used to assess calcification severity, and confirmed that all cases exhibited severe calcification even after RA/OA and required additional interventional treatment for calcified lesions. Appropriate case selection for combined treatment may be a key factor in achieving favorable outcomes. In this regard, on comparison of results with the stent expansion index using OCT, Dual-Prep demonstrated a better stent expansion index than the other treatment strategies. An inadequate stent expansion index suggests suboptimal stent expansion and is known to be an important determinant of long-term stent failure. Therefore, it is reasonable to consider that the favorable OCT analysis results of DUAL-Prep directly contributed to improved long-term outcomes. Thus, this study represents an outcome assessment of combined atherectomy and IVL therapy based on a pragmatic concept.

### Factors associated with MACE

Factors associated with MACE included baseline reference vessel diameter and postprocedural % stenosis. The incidence of MACE was significantly higher as both factors increased. This finding is partially consistent with the BENELUX-IVL registry, which reported clinical and technical predictors of MACE after coronary IVL. [20] In the BENELUX-IVL registry, multivariate analysis revealed that procedural complications, chronic total occlusion, in-stent restenosis, plaque modification after IVL, and greater post procedural residual stenosis were associated with MACE. Compared to their study, the present study used pre-IVL plaque modification in all cases, and excluded ISR cases. Furthermore, the in-hospital complication rate in this study was lower than that in the BENELUX-IVL Registry, with the proportion of ACS of 10.2% and 42%, respectively. These studies can be considered to have produced essentially similar results given the baseline differences in the populations studied. These findings reaffirm residual stenosis as a universal indicator and emphasize its importance as a procedural guide tool. Another factor is large vessels: luminal gain is often greater in larger vessels than small vessels, potentially leading physicians to consider the procedure adequate even when residual stenosis is suboptimal/inadequate. As vessel diameter increases, the thickness of calcification may also increase, resulting in more inadequate modification and consequently larger residual stenosis in large vessels. Notably, CN were not a determinant of outcome in this study, despite being a well-established risk factor for poor long-term prognosis after PC. Similarly, Ali et al. reported a pooled analysis using OCT data from the Disrupt CAD trials in which CN were identified in 18.7% (29/155) of lesions among 155 subjects, and IVL use resulted in equivalent stent expansion and lumen enlargement in both CN and non-CN lesions. [21] The Dual-Prep Registry OCT analysis yielded similar results, showing no difference in stent expansion based on CN presence. Adequate stent expansion is a fundamental requirement for favorable long-term outcomes, and is therefore likely reflected in the long-term freedom from MACE observed. Although no significant association was observed, the directionality of the association with MACE differed between eruptive CN and non-eruptive CN: eruptive CN showed a stronger association with MACE, consistent with findings suggested by previous reports. [22] However, due to the limited number of events, this finding should be interpreted with caution.

### Limitations

Several important limitations of this trial must be noted. First, this was an open-label, single-arm trial. Comparative studies based on hypotheses are needed to assess the superiority of this strategy over other treatment strategies. Second, the selection of treatment device size and subsequent therapy was left to the treating physician’s judgment and was not based on consistent rules. Third, the frequency of ACS was low, and it is unclear whether efficacy is consistent across all clinical situations. Fourth, while increased healthcare costs are a concern, the balance of cost-effectiveness remains unclear. Fifth, given the risk of overfitting due to the small number of events, multivariate analysis of event-related factors could not be performed, and caution should be exercised in interpreting this analysis. Therefore, these results require validation in future prospective studies. Finally, long-term prognosis over more than 1 year remains unclear.

## Conclusions

Atherectomy followed by IVL resulted in low 1-year rates of MACE, TLR, and stent thrombosis in patients with severely calcified coronary lesions. This approach may be considered for lesions where an “IVL-first” strategy is difficult to apply.

## Supplementary Information

Below is the link to the electronic supplementary material.

Supplementary file1

## Data Availability

Participant data from this clinical trial will not be shared without entering into contractual agreement.

## Abbreviations

CD-TVR: Clinically driven target vessel revascularization
CEC: Clinical evaluation committee
CI: Confidence interval
CN: Calcified nodule
DCB: Drug-coated balloon
DES: Drug eluting stent
IVL: Intravascular lithotripsy
MACE: Major adverse cardiac events
MI: Myocardial infarction
OA: Orbital atherectomy
OCT: Optical coherence tomography
OFDI: Optical frequency domain imaging
PCI: Percutaneous coronary intervention
RA: Rotational atherectomy
SCAI: Society for cardiovascular angiography and interventions
TLF: Target lesion failure
TLR: Target lesion revascularization
TVR: Target vessel revascularization

## References

[1] Gu D, Qu J, Zhang H, Zheng Z. Revascularization for coronary artery disease: principle and challenges. Adv Exp Med Biol. 2020;1177:75–100. 32246444 10.1007/978-981-15-2517-9_3

[2] Kirtane AJ, et al. Orbital atherectomy versus balloon angioplasty before drug-eluting stent implantation in severely calcified lesions eligible for both treatment strategies (ECLIPSE): a multicentre, open-label, randomised trial. Lancet. 2025;405:1240–51. 40174596 10.1016/S0140-6736(25)00450-7

[3] Stone GW, et al. Intravascular imaging vs angiography guidance for PCI of severely calcified lesions: the ECLIPSE trial. JACC Cardiovasc Interv. 2025;18:2338–51. 41093451 10.1016/j.jcin.2025.08.024

[4] Nakamura M, et al. Dual-Prep registry: atherectomy devices and intravascUlAr lithotripsy for the preparation of heavily calcified coronary lesions registry. Cardiovasc Interv Ther. 2025;40:553–64. 40354027 10.1007/s12928-025-01130-9 PMC12167257

[5] Ikari Y, Saito S, Nakamura S, Shibata Y, Yamazaki S, Tanaka Y, et al. Device indication for calcified coronary lesions based on coronary imaging findings. Cardiovasc Interv Ther. 2023;38:163–5. 36780124 10.1007/s12928-023-00914-1 PMC10020240

[6] Sakakura K, et al. Clinical expert consensus document on rotational atherectomy from the Japanese association of cardiovascular intervention and therapeutics: update 2023. Cardiovasc Interv Ther. 2023;38:141–62. 36642762 10.1007/s12928-022-00906-7 PMC10020250

[7] Thygesen K, et al. Fourth Universal Definition of Myocardial Infarction (2018). Circulation. 2018;138:e618–51. 30571511 10.1161/CIR.0000000000000617

[8] Abdel-Wahab M, et al. High-speed rotational atherectomy before paclitaxel-eluting stent implantation in complex calcified coronary lesions: the randomized ROTAXUS (Rotational Atherectomy Prior to Taxus Stent Treatment for Complex Native Coronary Artery Disease) trial. JACC Cardiovasc Interv. 2013;6:10–9. 23266232 10.1016/j.jcin.2012.07.017

[9] Chambers JW, et al. Pivotal trial to evaluate the safety and efficacy of the orbital atherectomy system in treating de novo, severely calcified coronary lesions (ORBIT II). JACC Cardiovasc Interv. 2014;7:510–8. 24852804 10.1016/j.jcin.2014.01.158

[10] Kawamoto H, et al. In-hospital and midterm clinical outcomes of rotational atherectomy followed by stent implantation: the ROTATE multicentre registry. EuroIntervention. 2016;12:1448–56. 27998836 10.4244/EIJ-D-16-00386

[11] Bouisset F, et al. Clinical outcomes of PCI with rotational atherectomy: the European multicentre Euro4C registry. EuroIntervention. 2020;16:e305–12. 32250249 10.4244/EIJ-D-19-01129

[12] Hajj M E, Maran A, Hill A, Staub S, Hajj S E, Fernandes V. One Year Outcomes of Orbital versus Rotational Atherectomy for the Treatment of Heavily Calcified Coronary Disease. *Interventional Cardiology* 2020; **122**.

[13] Genereux P, et al. Orbital atherectomy for treating de novo severely calcified coronary narrowing (1-year results from the pivotal ORBIT II trial). Am J Cardiol. 2015;115:1685–90. 25910525 10.1016/j.amjcard.2015.03.009

[14] Lee MS, Shlofmitz E, Goldberg A, Shlofmitz R. Multicenter registry of real-world patients with severely calcified coronary lesions undergoing orbital atherectomy: 1-year outcomes. J Invasive Cardiol. 2018;30:121–4. 29610442

[15] Kereiakes DJ, et al. Intravascular lithotripsy for treatment of severely calcified coronary lesions: 1-year results from the Disrupt CAD III study. J Soc Cardiovasc Angiogr Interv. 2022;1:100001. 39130140 10.1016/j.jscai.2021.100001 PMC11308114

[16] Saito S, et al. Intravascular lithotripsy for vessel preparation in calcified coronary arteries prior to stent placement- Japanese Disrupt CAD IV study 1-year results. Circ Rep. 2022;4:399–404. 36120480 10.1253/circrep.CR-22-0068 PMC9437473

[17] Sukul D, et al. Contemporary trends and outcomes of intravascular lithotripsy in percutaneous coronary intervention: insights from BMC2. JACC Cardiovasc Interv. 2024;17:1811–21. 38970579 10.1016/j.jcin.2024.04.039

[18] Butala NM, et al. Use of calcium modification during percutaneous coronary intervention after introduction of coronary intravascular lithotripsy. J Soc Cardiovasc Angiogr Interv. 2024;3:101254. 39132220 10.1016/j.jscai.2023.101254 PMC11308754

[19] van Oort MJH, et al. Current applications, procedural and 1-year outcomes of Rotatripsy for the treatment of calcified coronary lesions. Catheter Cardiovasc Interv. 2024;104:203–12. 38932584 10.1002/ccd.31140

[20] van Oort MJH, et al. Clinical and technical predictors of adverse cardiovascular events following coronary lithotripsy in the BENELUX-IVL registry. Am J Cardiol. 2026;258:261–7. 41135788 10.1016/j.amjcard.2025.10.018

[21] Ali ZA, et al. Outcomes of coronary intravascular lithotripsy for the treatment of calcified nodules: a pooled analysis of the Disrupt CAD studies. EuroIntervention. 2024;20:e1454–64. 39618263 10.4244/EIJ-D-24-00282 PMC11586657

[22] Shin D, et al. Calcified nodule in percutaneous coronary intervention: therapeutic challenges. JACC Cardiovasc Interv. 2024;17:1187–99. 38811101 10.1016/j.jcin.2024.03.032

