# Supplementary material for: Dual-Prep registry: Atherectomy devices and intravascUlAr lithotripsy for the PREParation of heavily calcified coronary lesions registry, 1-year results
Source: Cardiovasc Interv Ther. 2026 Mar 19;41(3):530–9. doi: 10.1007/s12928-026-01264-4 (PMC13279709; doi:10.1007/s12928-026-01264-4)
Supplement: Supplementary file 1 — Supplementary file1 [file 12928_2026_1264_MOESM1_ESM.docx]

Supplementary files

Figure S1: Post-procedure antiplatelet therapy

Figure S2: Kaplan-Meier curve for each component of MACE

Table S1: MACE incidence in various subgroups

Figure S1.


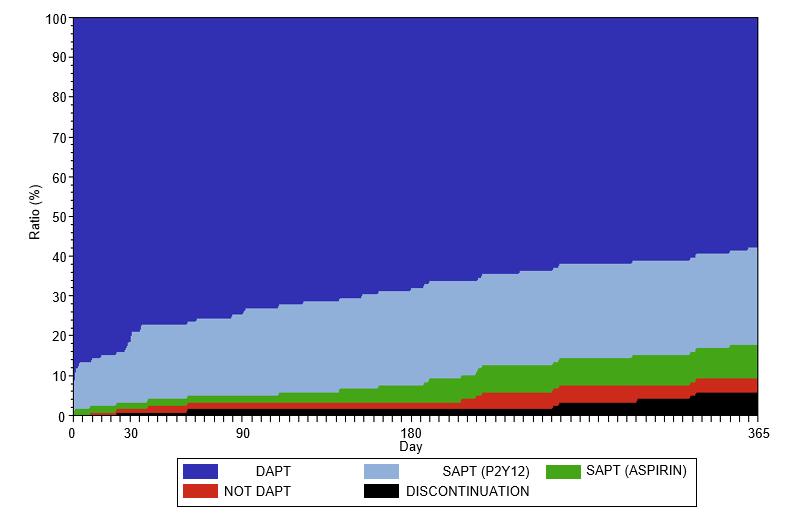


Figure S2.

Cardiac death


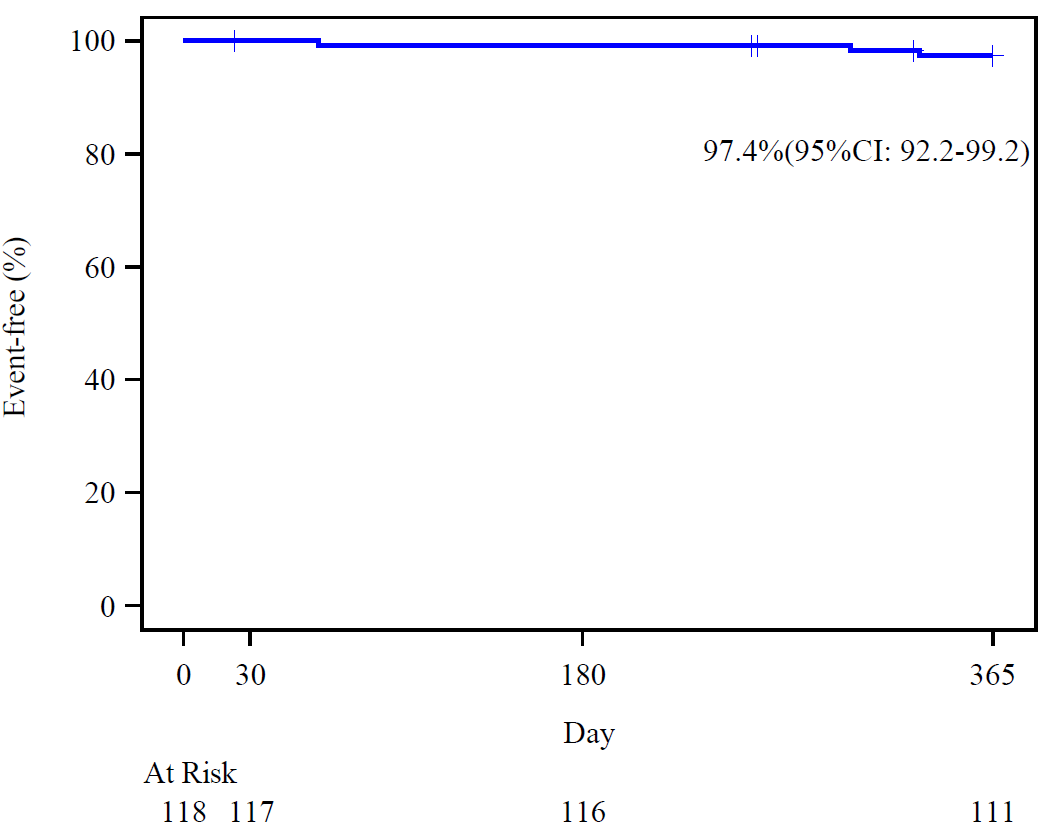


MI


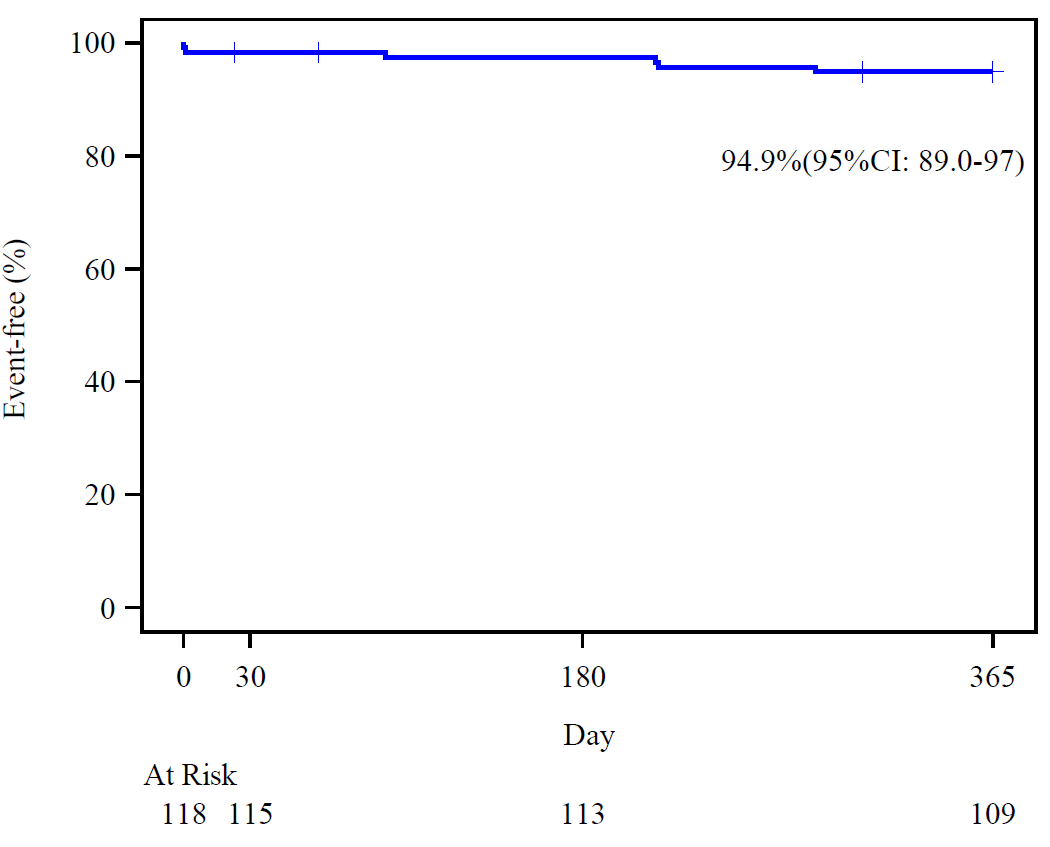

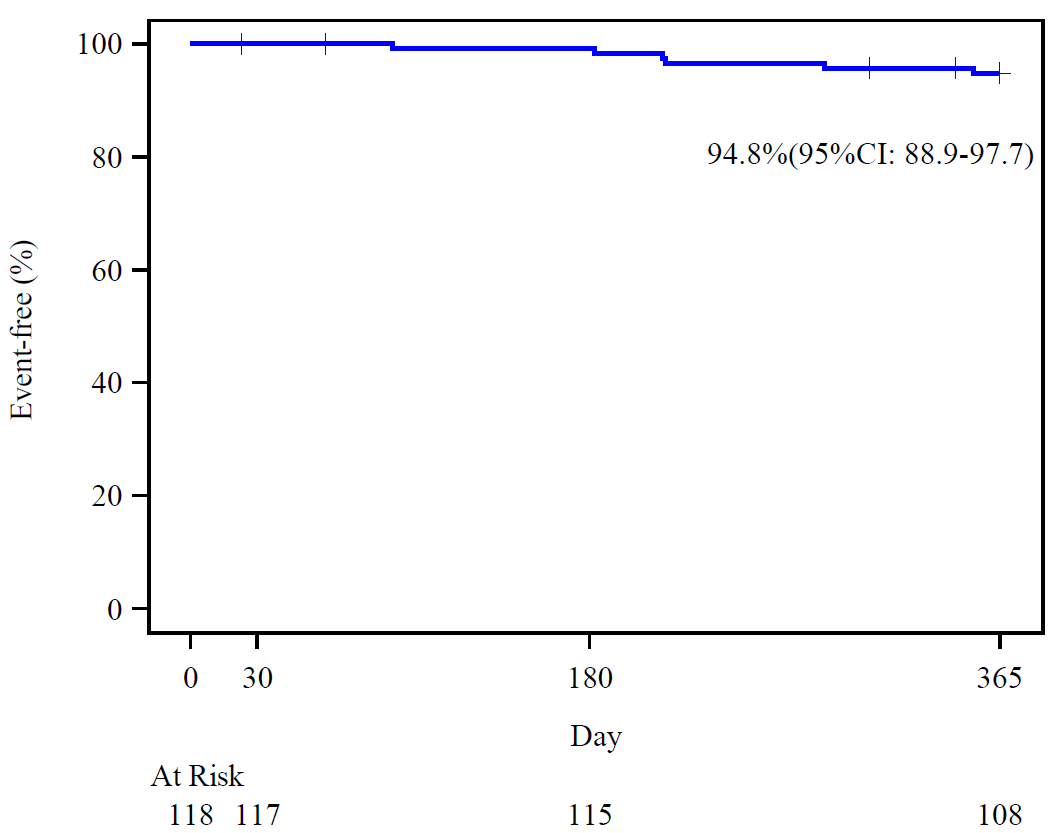


Clinical driven TLR

Table S1.

|  | Overall  (n=118) | MACE  (n=9) | MACE-free  (n=109) |
| --- | --- | --- | --- |
| Age | 75.8±8.9 | 81.2±9.8 | 75.4±8.75 |
| Male | 83(70.3) | 4 (44.4) | 79 (72.5) |
| Body weight (kg) | 60.3±12.9 | 57.26±14.06 | 60.53±12.82 |
| Diabetes mellitus | 67 (56.8) | 7 (77.8) | 60 (55.0) |
| Hyperlipidemia | 91 (77.1) | 5 (55,6) | 86 (78.9) |
| Smoking | 59 (50.0) | 5 (55.6) | 54 (49.5) |
| Hemodialysis | 30 (25.4) | 4 (44.4) | 26 (23.9) |
| eGFR (n=117) | 45.10±27.01 | 31.06±27.61 | 46.27±26.75 |
| LAD | 75 (63.6) | 4 (44.4) | 71 (65.1) |
| Ostial lesion | 77 (65.3) | 7 (77.8) | 70 (64.2) |
| Ref (mm) (n=117) | 2.67±0.69 | 3.20±1.03 | 2.66±0.67 |
| Pre MLD (mm) (n=117) | 0.72±0.28 | 0.86±0.50 | 0.70±0.26 |
| Pre %DS (n=117) | 72.6±9.6 | 73.45±9.37 | 72.48±9.63 |
| Post MLD (mm) | 2.67±0.57 | 2.81±0.67 | 2.65±0.56 |
| Post %DS | 15.85±5.62 | 21.20±6.45 | 15.41±5.35 |
| Burr-to-artery ratio (n=98) | 0.62±014 | 0.55±0.18 | 0.63±0.14 |
| IVL/vessel size (n=117) | 1.17±0.23 | 1.11±0.29 | 1.18±0.23 |
| Stent size/vessel size (n=117) | 1.23±0.25 | 1.11±0.20 | 1.24±0.26 |
| Calcium nodule eruptive | 29 (24.6) | 4 (44.4) | 25 (22.9) |
| Calcium nodule non-eruptive | 31(26.3) | 1 (11.1) | 30 (27.5) |
| Stent expansion index (n=107) | 81.11±14.88 | 82.27±9.02 | 81.01±15.34 |
| Asymmetry index (n=107) | 0.39±0.15 | 0.40±0.15 | 0.39±0.15 |
| Eccentricity index (n=107) | 0.72±0.09 | 0.70±0.09 | 0.72±0.09 |
| Minimum stent area (mm^2^) (n=107) | 5.53±2.12 | 6.43±2.19 | 5.45±2.11 |
